# Mentalizing and Information Propagation through Social Network: Evidence from a Resting-State-fMRI Study

**DOI:** 10.3389/fpsyg.2016.01716

**Published:** 2016-11-04

**Authors:** Huijun Zhang, Lei Mo

**Affiliations:** Guangdong Provincial Key Laboratory of Mental Health and Cognitive Science, Center for Studies of Psychological Application, School of Psychology, South China Normal UniversityGuangzhou, China

**Keywords:** microblog, information propagation, social sharing, emotion, mentalizing, rs-fMRI, functional connectivity

## Abstract

Microblogs is one of the main social networking channels by which information is spread. Among them, Sina Weibo is one of the largest social networking channels in China. Millions of users repost information from Sina Weibo and share embedded emotion at the same time. The present study investigated participants’ propensity to repost microblog messages of positive, negative, or neutral valence, and studied the neural correlates during resting state with the reposting rate of each type microblog messages. Participants preferred to repost negative messages relative to positive and neutral messages. Reposting rate of negative messages was positively correlated to the functional connectivity of temporoparietal junction (TPJ) with insula, and TPJ with dorsolateral prefrontal cortex. These results indicate that reposting negative messages is related to conflict resolution between the feeling of pain/disgust and the intention to repost significant information. Thus, resposting emotional microblog messages might be attributed to participants’ appraisal of personal and recipient’s interest, as well as their cognitive process for decision making.

## Introduction

Microblogging is a popular social medium for people to seek or share information. Imagine that a user writes a microblog message (no more than 140 characters) and posts it to a number of friends on the web; at the same time, he or she may surf across hundreds of messages posted by friends or broadcasters (e.g., posts made by the government, media, or celebrities). The user can select some interesting activities, opinions, and viewpoints to comment on or further repost them through the Internet. That is the way that we seek and share information via social media today. In China, microblogs are increasingly popular in interpersonal communication and have brought a dramatic change to patterns of information diffusion (data from China Internet Network Information Center [CINI], 2016a,b). As of December, 2014, over 249 Million Chinese people had accounts on the largest microblog system in China, Sina Weibo (weibo^1^). They produce over 90 million posts in Sina weibo per day (from China Internet Network Information Center [CINI], 2015).

Reposting is the step in which a microblog message is propagated. It is the key activity that characterizes microblogging as a new type of social medium of information dissemination. From among a massive amount of information obtained via friends and followed publishers, an active user can select pieces of microblog messages and further repost them in a short time. Through this activity, each user serves as an information recipient, producer and propagator at the same time (Java et al., 2007; Kwak et al., 2010). In contrast to conventional means of communication, which require plenty of time for diffusion, microblog messages spread information effectively and efficiently via divergent reposts (China Internet Network Information Center [CINI], 2016b).

Several studies have investigated reposting behavior on the web or in a lab environment. One study used self-report questionnaires and daily diaries to statistics the frequency of sharing emotion information in all types of social media (Choi and Toma, 2014). Falk et al. (2012, 2013) employed functional magnetic resonance imaging (fMRI) as a method to explore multiple simultaneous processes and neurocognitive networks during information or idea exposure, revealing implicit and explicit factors engaged in social communication that might not be conspicuous from self-report measures or behavioral tasks. Their results indicate that individual’s intention to spread an idea is based on their evaluation toward self-interest and a prediction to the recipient’s interest. Thus, the self-relevance processing system that consists of medial prefrontal cortex (MPFC) and posterior cingulate cortex (PCC)/Precuneus and the mentalizing regions that consists of dorsomedial prefrontal cortex (DMPFC) and temporoparietal junction (TPJ) are engaged in the process of information propagation. Besides, reward system is activated because information sharing is a prosocial activity, which gives us the same positive experience as what we feel when given rewards (Falk et al., 2013).

The self-relevance processing system is involved in tasks that evoke self-relatedness (Tacikowski et al., 2013), self-reflection (Johnson et al., 2006), and self-judgment (Kelley et al., 2002), demonstrating its important role in evaluating self-relatedness and self-interest for information propagation (Falk et al., 2013). A recent study observed intrinsic activity at this region was associated with participants’ degree of self-related information sharing through social network, e.g., the self-reported frequency of updating personal profile information or status on Facebook. The functional connectivity between MPFC and dorsolateral prefrontal cortex (DLPFC) was correlated with the self-related sharing scores calculated from updating frequency on Facebook (Meshi et al., 2016). This study shows not only the task-related neural activity at self-relevance processing system can predict participant’s intention of sharing self-related information, the spontaneous activity at these regions, especially at MPFC is also associated with participant’s preference in information diffusion, reflecting an evaluation to self-relatedness and self-interest.

The mentalizing system is involved in a cognitive process to reason other’s mental state (Bzdok et al., 2012; Schurz et al., 2014). The volume of the brain regions within this system is correlated to the online social networking services (SNS) size (Kanai et al., 2011). Increased activity at these regions is observed when participants are engaged in mentalizing or theory of mind (TOM) task (Hyatt et al., 2015). This activity can further predict individual’s behavioral performance in such task (Kanske et al., 2015). Subramani and Rajagopalan (2003) propose that the ability of the recommender to accurately predict the recipient’s interests and preferences is essential for information propagation in online social network. We might speculate neural activity at TPJ reflects participants’ consideration to the recipient’s interest when they are making a decision about reposting a microblog message or not. One recent study showed the decreased functional connectivity between TPJ and supramarginal gyrus in autism patients was paralleled with their deficiency in mentalizing (Hoffmann et al., 2016), indicating the resting state functional connectivity (rs-functional connectivity) between TPJ and other brain regions is associated with individual’s mentalizing ability. Therefore, we may further speculate the rs-functional connectivity between mentalizing system, e.g., TPJ and other brain regions would be also associated with participant’s ability to predict the recipient’s interests in information propagation.

Accompanied with reposting microblog messages, the embedded emotion in microblog is spontaneously shared. Empirical evidence shows that emotional information is more likely to be propagated than the neutral information obtained through conventional media (Poels and Dewitte, 2006) and online networks (Berger and Milkman, 2012; Hidalgo et al., 2015). Sharing an emotional event (e.g., through facial expression or an emotional message) elicits hyperactivity in the reward system (Schilbach et al., 2010; Falk et al., 2012, 2013). That means displaying and sharing emotion plays an important role in social communication. For example, sharing negative information attracts more attention from the public and benefits our survival (Pratto and John, 1991; Baumeister et al., 2001); on the other hand, positive information may boost other users’ positive feelings (Otto et al., 2015) and neural activity at mentalizing regions (Falk et al., 2012). These results indicate that sharing information with different emotional valence might be associated with neural activity at distinct brain regions.

In real-life SNS, microblog messages are displayed in different formats. Some of them are highlighted with conspicuous title, others were accompany with remarkable pictures. These confounding factors would make the microblog messages hyperarousal or blurry. In this study, we would exclude the confounding titles and pictures, and balance the arousal and familiarity of each type of experimental materials. Thus, these experiment materials would be revised into a standardized format and read by participants in lab.

In the present study, we would like to investigate the relationship between rs-functional connectivity with regions engaged in microblog repost and individual’s preference to microblog with different emotional valence. Here, we used standardized microblog messages as experimental materials. The number of reposts of microblog messages can quantitatively reflect participants’ propensity to microblog messages with different emotional valence in reposting (Falk et al., 2013). The rs-functional connectivity between brain regions can serve as indexes of corresponding ability and cognitive process. Based on pilot data, we hypothesized that the microblog messages with emotional valence would be discriminatively reposted. Negative messages would be more likely to repost. Moreover, the reposting rate of negative messages would be correlated with the functional connectivity between the mentaling regions of interest, i.e., TPJ and DMPFC, and the brain regions engaged in negative processing (e.g., insula). The results would be helpful in uncovering preferences for information propagation and emotion contagion through social media, as well as the neural activity associated with the corresponding psychological processes.

## Materials and Methods

### Participants

We recruited 28 right-handed Chinese University students (14 males and 14 females). They ranged from 18 to 25 years old (four participants did not report their age; the mean age of 24 participants = 21.21 years ± 1.82). All participants had finished at least high school (i.e., they each had at least 12 years of formal schooling). They each had their own microblog (Weibo) account and had experience reading and reposting information through social media. All of them had normal or corrected-to-normal vision. None had a history of traumatic brain injury, medical conditions, or any psychiatric disorder that could affect neural activity and brain functioning.

This study was approved by the Human Research Ethics Committee for Non-Clinical Faculties in South China Normal University. All participants signed on a consent form which exhibited the study purpose, the experimental procedure and the amount of payment in the beginning of the study. They were allowed to quit at any stage of the experiment without any punishment.

### Materials and Experimental Task

Across three categories, 120 pieces of microblog messages were extracted from Sina Weibo microblog system (Weibo^2^). The microblog messages in the positive category reported positive events in social life (e.g., someone was praised by the public for his/her contribution), whereas the microblog messages in the negative category reported negative events (e.g., a terrible accident happened, killing dozens of students). The microblog messages with no emotional significance were categorized as neutral and were included to provide a more realistic simulation of microblog messages. Each piece of microblog message was revised or modified to be a standard length (90–140 Chinese characters), and was written in the third person (as is commonly used in the newspaper). In order to control for possible bias from the original poster, the microblog messages used in this study were posted by four different publishers: Tencent, Sina, Sohu, and NetEase (all common, official publishers in Sina Weibo microblog).

Thirty participants were recruited to rate the microblog messages on (1) the emotional valence shown by each piece of microblog message, (2) the emotional arousal elicited by each piece, and (3) their familiarity with the microblog message provided in each piece (all on 7-point scales). Using these ratings, 90 out of the initial 120 pieces of microblog messages were selected (30 in each category). The three categories of microblog messages differed significantly in valence [*F*(2,87) = 380.52, *p* < 0.001] and arousal [*F*(2,87) = 124.07, *p* < 0.001], but not in familiarity (*p* > 0.1, see **Table 1**). *Post hoc* analyses showed that the valence of all three categories differed significantly from each other (*p*s < 0.001). In addition, neutral microblog messages elicited less arousal than both positive microblog messages [*t*(58) = 12.15, *p* < 0.001] and negative microblog messages [*t*(58) = 14.13, *p* < 0.001], whereas no significant difference in arousal was observed between positive and negative microblog messages [*t*(58) < 1]. In order to exclude a familiarity effect, pieces of microblog messages rated higher than 4.5 (out of 7) on the familiarity scale were excluded. The three categories of microblog messages did not differ in length [*F*(2,87) = 1.37, *p* = 0.258].

**Table 1:** Average ratings toward three categories of microblog messages and average length.

	Valence	Arousal	Familiarity	Length (characters)
Positive	5.22 ± 0.32	5.12 ± 0.27	2.29 ± 0.59	109.27
Negative	2.24 ± 0.58	5.08 ± 0.39	2.43 ± 0.82	111.33
Neutral	3.97 ± 0.30	3.80 ± 0.43	2.05 ± 0.51	107.33

*Three categories of microblog messages significantly differed in the rating of valence. Neutral microblog messages were rated lower arousal than positive and negative microblog messages. All categories did not differed in ratings of familiarity and length.*

Participants read all microblog messages after resting state fmri scanning in the scanner. Each trial began with a fixation cross in the center of the screen for 1 s. A piece of microblog message was then presented on the screen for 15 s. This duration was based on the average reaction time from participants in the pilot study. The screen layout mimicked the interface of the Sina Weibo microblog. In a subsequent response stage, the participants’ task was to make a prompt decision (within 3 s) to repost or not repost this piece of microblog message. The decisions “repost” and “not-repost” corresponded to two buttons both on response pad and on screen (one on the left and one on the right, counterbalanced). After the decision was made, feedback was given in the form of the words “Reposted” or “Not Reposted” appearing on the screen for 1 s. The inter-stimulus interval was jittered between 3 and 6 s.

In total, each participant read 90 pieces of microblog messages (all microblog messages were listed in Supplementary Materials). All microblog messages were pseudorandomized within each participant such that no more than three pieces of microblog messages with the same valence were presented successively. The total duration of the task was 36 min.

### Image Acquisition, Preprocessing, and Statistical Analysis

Magnetic resonance imaging (MRI) data were acquired using a Siemens Tim Trio 3T MRI scanner at South China Normal University. The scanner was equipped with a standard eight-channel head coil. Functional images were acquired based on a T2^∗^-weighted gradient-echo echo-planar imaging pulse sequence parallel to the AC-PC plane with following parameters: 160 volumes, 36 slices in each volume, TR = 3000 ms, TE = 30 ms, slice thickness = 3.5 mm, flip angle = 90°, matrix = 64 × 64, and a FOV = 224 mm × 224 mm. Anatomical images were obtained based on a T1-weighted gradient-echo pulse sequence (176 slices, TR = 1900 ms, TE = 2.52 ms) with a spatial resolution of 1 mm × 1 mm × 1 mm.

Preprocessing of the imaging was carried out by SPM8 software (Wellcome Department of Cognitive Neurology, London, UK), DPARSFA toolbox Version 3.0 (Yan and Zang, 2010), and DPABI toolbox (Yan et al., 2016) implemented in Matlab R2012a (Version 7.14.0.739, MathWorks Inc., Sherborn, MA, USA). The first 10 volumes of rs-fMRI data were discarded and the preserved functional images were corrected to the middle slice in each volume in order to adjust the differences in acquisition times of multiple slices. They were realigned to the first volume in the resting state scanning session for head motion correction and resliced to form a mean image. They were then co-registered to high-resolution T1 images at an individual level. After that, six rigid body head motions parameters, white matter, CSF and global signal of the whole brain were regressed out (Yan et al., 2013). Functional images were normalized to the Montreal Neurological Institute (MNI) template (resolution = 3 mm × 3 mm × 3 mm) using unified segmentation T1 images and smoothed with a Gaussian kernel (FWHM = 8 mm) to decrease the individual differences and increase the normality of signals. The smoothed signals were detrended and band-pass filtered (0.1–0.01 Hz) to remove the cardiac and respiratory signals before they were ready for further analysis.

Priori regions of interest (ROIs, 8 mm spheres) including bilateral TPJ (MNI coordinates of center: -48, -51, 15; and 51, -60, 18), DMPFC (MNI coordinates of center: -12, 42, 37; and 11, 39, 43) and MPFC (MNI coordinates of center: -3, 51, -3) from Falk et al. (2012, 2013) were engaged as seeds separately for functional connectivity analyses. The mean neural activities of all voxels within the corresponding seed ROIs for each participant were extracted and correlated (using Pearson’s correlation, *r*) with all other brain voxels for every time point within the time course of the preprocessed rs-fMRI data. *R*-values were then transformed to approximately normally distributed Fisher’s *Z* scores to produce the standardized functional connectivity (*z*-fc) maps at individual level. The *z*-fc maps for all participants were entered into second-level, voxel-wise, multiple regression models for identifying the regions where we could observe significant correlations between *z*-fc and reposting rate of each type. Results were corrected for multiple comparisons using Gaussian Random Field theory (GRF: voxel threshold: |*Z*| > 2.32, cluster-level threshold: *p* < 0.05). The mean functional connectivity of all voxels within each activated cluster was further extracted to calculate the magnitude of correlation between rs-fMRI and reposting rate of corresponding type of microblog messages.

## Results

### Behavioral Results

A one-way ANOVA was conducted to explore the difference in reposting rate associated with emotional valence. A significant difference was observed across three valences of microblog messages, *F*(2,54) = 18.19, η^2^ = 0.40, *p* < 0.001. Microbologs with positive valence (*M* = 0.48, *SD* = 0.24) were less reposted than those with negative valence (*M* = 0.64, *SD* = 0.21, Mean Difference = -0.16, Bonferroni corrected *p* = 0.012) but more reposted than neutral ones (*M* = 0.30, *SD* = 0.28; Mean Difference = 0.18, Bonferroni corrected *p* = 0.005).

### Seed-Based Functional Connectivity Results

Functional connectivity was computed based on seeds at bilateral TPJ, DMPFC, and MPFC. The correlations between average functional connectivity with reposting rate of each type of microblog messages were compared with zero, respectively.

**FIGURE 1:**
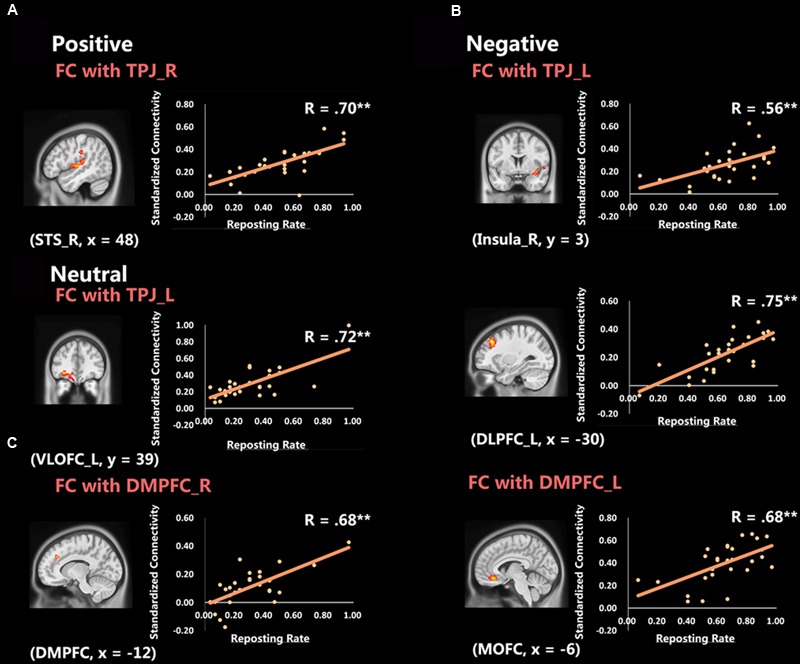
**rs-fcMRI results for mentalzing seeds [Gaussian Random Field theory (GRF) corrected voxel threshold: | Z| > 2.32, cluster-level threshold: *p* < 0.05]. (A)** Significant positive correlation between functional connectivity with right temporoparietal junction (TPJ) and reposting rate of positive microblogs in right STS. **(B)** Significant positive correlations between functional connectivity with left TPJ and reposting rate of negative microblogs in right insula and left DLPFC (middle frontal lobe), and significant positive correlation between functional connectivity with left dorsomedial prefrontal cortex (DMPFC) and reposting rate of negative microblogs in bilateral MOFC. **(C)** Significant positive correlation between functional connectivity with left TPJ and reposting rate of neutral microblogs in left VLOFC, and significant positive correlation between functional connectivity with right DMPFC and reposting rate of neutral microblogs in DMPFC.

Significant correlation with reposting rate of positive microblog messages was observed for the functional connectivity between right TPJ (ROI center at 51, -60, 18, MNI coordinates) and right superior temporal lobe (BA 48, peak at 48, -15, 3, *Z* = 3.87), *R* = 0.70, *p* < 0.001 [see **Figure 1A**], indicating participants with larger connectivity between these two regions would repost more positive microblog messages. Significant positive correlations with reposting rate of negative microblog messages were observed for the functional connectivity between left TPJ (ROI center at -48, -51, 15) and left middle frontal lobe (BA 9, peak at -30, 30, 39, *Z* = 4.33), *R* = 0.75, *p* < 0.001, and right insula (BA 48, peak at 39, 3, -9, *Z* = 3.43), *R* = 0.56, *p* < 0.001, as well as the functional connectivity between left DMPFC (ROI center at -12, 42, 37) and MOFC (BA 11, peak at -6, 33, -15, *Z* = 4.01), *R* = 0.59, *p* < 0.001 [see **Figure 1B**]. Significant positive correlations with reposting rate of neutral microblog messages were observed for the functional connectivity between left TPJ (ROI center at -48, -51, 15) and left VLOFC (BA 11, peak at -30, 39, -9, *Z* = 4.02), *R* = 0.72, *p* < 0.001, as well as the functional connectivity between bilateral DMPFC (BA 32, peak at -12, 33, 33, *Z* = 3.93), *R* = 0.68, *p* < 0.001 [see **Figure 1C**]. All significantly correlated regions are shown in **Table 2** and **Figure 1**.

**Table 2:** rs-fcMRI results for mentalizing seeds [Gaussian Random Field theory (GRF) corrected voxel threshold: | Z| > 2.32, cluster-level threshold: *p* < 0.05].

	Cluster-level		Peak-level			MNI coordinates (mm)		
	*p* (unc)	*k*	*p* (unc)	*T*	*Z*	*x*	*y*	*z*
**(A) Positive**								
**Right TPJ**								
Right Superior	0.001	242	0.000	4.55	3.87	48	-15	3
Temporal Lobe			0.000	4.05	3.53	51	-3	3
(BA 48)			0.000	3.73	3.31	63	12	15
**(B) Negative**								
**Left TPJ**								
Left Middle	0.000	252	0.000	5.31	4.33	-30	30	39
Frontal Lobe			0.000	3.94	3.46	-48	33	3
(BA 9)			0.000	3.81	3.36	-36	27	12
Right Insula	0.000	273	0.000	3.90	3.43	39	3	-9
(BA 48)			0.000	3.82	3.37	51	-6	0
**Left DMPFC**								
Left MOFC	0.003	167	0.000	4.77	4.01	-6	33	-15
(BA 11)			0.001	3.55	3.18	-15	27	-15
			0.001	3.38	3.05	9	33	-15
**(C) Neutral**								
**Left TPJ**								
Left VLOFC	0.001	260	0.000	4.79	4.02	-30	39	-9
(BA 11)			0.000	4.11	3.58	-24	51	-15
			0.000	3.76	3.33	-24	30	-9
**Right DMPFC**								
Left DMPFC								
(BA 32)	0.000	274	0.000	4.65	3.93	-12	33	33

***(A)** Clusters with significant positive correlations between functional connectivity with right TPJ and reposting rate of positive microblog messages. **(B)** Clusters with significant positive correlations between functional connectivity with Left TPJ and reposting rate of negative microblog messages, and clusters with significant positive correlations between functional connectivity with left DMPFC and reposting rate of negative microblog messages. **(C)** Clusters with significant positive correlations between functional connectivity with Left TPJ and reposting rate of neutral microblog messages, and clusters with significant positive correlations between functional connectivity with right DMPFC and reposting rate of neutral microblog messages.*

## Discussion

The idiom “For evil news rides fast, while good news baits later” indicates a phenomenon in which bad news receives higher publicity than good news. The same case has been observed in microblogs. In the present study, we investigated the behavioral patterns of opinion propagation through microblog messages and rs-functional connectivity of related brain regions. In line with our hypothesis, negative messages were propagated more frequently compared with positive messages and neutral messages. The brain regions involved in mentalizing were associated with individual differentiated preference of emotional valence. These evidence reveal an important behavioral pattern in which negative information prevailed in the transmission through the social media, and helped to identify the neural correlates of this process. This result extends our knowledge of the reposting behavior in microblogs and the corresponding intrinsic neural connectivity.

### The Prevalence of Negative Microblog Messages

Sharing information is a productive activity in the social network. Some previous studies show people are thought to have higher intention to share the “good news” with peers (Gable and Reis, 2010; Choi and Toma, 2014) because it improves the individual’s well-being and the atmosphere in a group (Otto et al., 2015). However, our behavioral findings indicate that positive and neutral news tend to be ignored by microblog users, while negative microblog messages are more frequently reposted. Negative stimuli exert a larger effect in individuals’ cognitive performance and attention (Ito et al., 1998; Ohira et al., 1998), helping them avoid dangers (Neuberg et al., 2011), and thereafter robustly attract participants’ interest and are more likely to be propagated than other emotion in SNS. These findings are comparable to those in recent studies on the prevalence of “bad news.” People are more interested in negative gossip about celebrities (e.g., the president did the wrong thing) and positive gossip about themselves. In previous research, hearing negative gossip about others induce higher emotion ratings, as well as greater neural activity in the reward system (Takahashi et al., 2009). Moreover, our findings further extend the boundary of existing knowledge of emotion contagion through SNS. People are not only attracted by negative messages but also were eager to distribute them, making the bad news travel fast.

### Functional Connectivity of TPJ and Other Brain Regions Associated with the Reposting Rate of Negative Microblog Messages

As for rs-fMRI, TPJ is one of key node of mentalizing network (Mars et al., 2012). It plays a key role in social communication (O’Donnell et al., 2015; Tang et al., 2016), even in the implicit condition (Zerubavel et al., 2015; Welborn et al., 2016). In the conditions without face-to face communication, activity at TPJ was highly increased when participant engaged in more consideration to other’s perspective (Falk et al., 2013). This hyperactivity helps participant successfully spread his/her own idea to others. In another study, the amplified activity at this system can predict participant’s usage of social language to expose one’ own thought (O’Donnell et al., 2015). Our result further extends these results and demonstrates that TPJ also engaged in mentalizing, an essential process of information propagation through on-line SNS. The increased reposting rate of negative messages is parallel with increased rs-functional connectivity of TPJ with insula and with middle frontal lobe.

Insula is the key node of empathy, especially the feeling of other’s pain (meta analysis from Fan et al., 2011; Lamm et al., 2011). Kanske et al. (2015) propose the distinct functions of insula and TPJ: the former underlie empathy, while the latter is involved in mentalizing or theory of mind. Since our messages in negative category report negative events in social life, the functional connectivity between these two regions might be associated with individual selective ability to feel other’s pain emotionally and cognitively. The increased functional connectivity reflects participants’ higher sensitivity to the pain of character described in negative messages, leading to their higher tendency to reposting such type of messages. Another possibility of positive correlation between the reposting rate of negative messages and rs-functional connectivity of TPJ and insula reflects participants’ sensitivity to disgust stimuli exposed by negative messages. Participants who exhibit a higher propensity of disgust both from their own and from recipient’s perspectives (Phillips et al., 1997) would be more likely to repost this type of messages due to its social significance.

It is worth noticing that increased rs-functional connectivity between TPJ with middle frontal lobe was also observed. More than evaluating the emotional significance of microblog messages from self and other’s perspectives, our brains are also preparing for future successful social interactions by considering the social significance of message. Thus, the cognitive control network is engaged in reposting negative messages due to its significant effect on the survival of human beings. This regulation conflicts with an intuitive process that represents self-interest and motivation (Feng et al., 2015), in which making decisions needs extra cognitive resources to resolve the ambivalence, inducing higher activity at bilateral DLPFC (Cikara et al., 2010; Kennerley and Walton, 2011). Moreover, it is demonstrated that activity at TPJ not only reflects participant’s consideration to other’s benefit, it is also related to the conflict resolution of benefits between self and others (Strombach et al., 2015). Our findings indicate participants are involved in a similar regulating process when participants are sharing on the web rather than face-to-face. The microblog messages that make us to undergo a feeling of pain disgust or pain might be those with significant social effects need to be reposted to the public. Participants with higher sensitivity to negative stimuli would have a higher tendency to intuitively avoid them but with have higher consciousness to its significance, so the simultaneous hyperactivity at these two regions leads to the increased rs-functional connectivity between TPJ with middle frontal lobe which compensates the increased rs-functional connectivity between TPJ with insula, as well as the increased reposting rate. This shows the reason why negative messages are given priority in the information sharing on the microblog.

### Limitation

Firstly, the relationships between neural activity and emotional valence have eluded consensus (Lamm et al., 2015; Wagner et al., 2015). In this study, we only measured rs-fMRI and reposting rate of each emotional type in lab, exploring the association between rs-functional connectivity and reposting rate. It is far from drawing a conclusion on the neural basis of reposting behavior in real-life SNS. Task-related fMRI study would be further conducted to measure the neural activity induced by microblog messages with different valence and related to information propagation. On the other hand, the study investigating the association between neural activity and behavioral performance in real-life by excluding confounding factors is also needed.

Second, microblog messages are restricted to one period of time, which might induce different emotional arousal and familiarity to the participants who participate in earlier stage and the participants who participate in later stage. Thus in this study, there were only 28 participants recruited within 1 month. The number is relatively small in imaging study exploring functional connectivity. Further studies with larger sample size may be conducted by better selection and controlling the experimental materials that are not time-restricted.

Third, we explored the functional connectivity of self-reference processing system and mentalizing system but we observed no significant correlation between the functional connectivity of self-reference processing regions and reposing rate. The discrepancy between our result and previous study might be due to the difference in measures and materials. Meshi et al. (2016) calculated the self-reported sharing score by participant’s self-reported updating frequency on Facebook, which might be more related to participant’s self-construct. We calculated the reposing rate of microblog messages, which described almost social events. This selection of our materials decreased its relatedness to self. Further analysis would be conducted for comparing the distinct neural associates with self and social sharing.

## Author Contributions

HZ was responsible for data analysis and writing this manuscript. LM was responsible for supervision and result interpretation. All authors agree to be accountable for the content of the work.

## Conflict of Interest Statement

The authors declare that the research was conducted in the absence of any commercial or financial relationships that could be construed as a potential conflict of interest.

## Footnotes

1 www.weibo.com

2 www.weibo.com

## References

[B1] Baumeister R. F. Bratslavsky E. Finkenauer C. Vohs K. D. (2001). Bad is stronger than good. *Rev. Gen. Psychol.* 5 323–370.

[B2] Berger J. Milkman K. L. (2012). What makes online content viral? *J. Mark. Res.* 49 192–205. 10.1509/jmr.10.0353

[B3] Bzdok D. Schilbach L. Vogeley K. Schneider K. Laird A. R. Langner R. (2012). Parsing the neural correlates of moral cognition: ALE meta-analysis on morality, theory of mind, and empathy. *Brain Struct. Funct.* 217 783–796. 10.1007/s00429-012-0380-y 22270812 PMC3445793

[B4] China Internet Network Information Center [CINI] (2015). *The 35th Statistical Report on Internet Development in China.* Available at: http://cnnic.com.cn/IDR/ReportDownloads/201604/P020160419390562421055.pdf

[B5] China Internet Network Information Center [CINI] (2016a). *The 37th Statistical Report on Internet Development in China.* Available at: http://www1.cnnic.cn/IDR/ReportDownloads/201604/P020160419390562421055.pdf

[B6] China Internet Network Information Center [CINI] (2016b). *Social Netwoking in China Internet Network Information Center [CINI], 2015*. Available at: http://www.cnnic.cn/hlwfzyj/hlwxzbg/sqbg/201604/P020160408334860042447.pdf

[B7] Choi M. Toma C. L. (2014). Social sharing through interpersonal media: patterns and effects on emotional well-being. *Comput. Hum. Behav.* 36 530–541. 10.1016/j.chb.2014.04.026

[B8] Cikara M. Farnsworth R. A. Harris L. T. Fiske S. T. (2010). On the wrong side of the trolley track: neural correlates of relative social valuation. *Soc. Cogn. Affect. Neurosci.* 5 404–413. 10.1093/scan/nsq011 20150342 PMC2999760

[B9] Falk E. B. Morelli S. A. Welborn B. L. Dambacher K. Lieberman M. D. (2013). Creating buzz: the neural correlates of effective message propagation. *Psychol. Sci.* 24 1234-1242 10.1177/0956797612474670 23722983

[B10] Falk E. B. O’Donnell M. B. Lieberman M. D. (2012). Getting the word out: neural correlates of enthusiastic message propagation. *Front. Hum. Neurosci.* 6:313 10.3389/fnhum.2012.00313 PMC3506032 23189049

[B11] Fan Y. Duncan N. W. de Greck M. Northoff G. (2011). Is there a core neural network in empathy? An fMRI based quantitative meta-analysis. *Neurosci. Biobehav. Rev.* 35 903–911. 10.1016/j.neubiorev.2010.10.009 20974173

[B12] Feng C. L. Luo Y. J. Krueger F. (2015). Neural signatures of fairness-related normative decision making in the ultimatum game: a coordinate-based meta-analysis. *Hum. Brain Mapp.* 36 591–602. 10.1002/hbm.22649 25327760 PMC6869807

[B13] Gable S. L. Reis H. T. (2010). “Good news! Capitalizing on positive events in an interpersonal context,” in *Advances in Experimental Social Psychology* Vol. 42 ed. Zanna M. P. (San Diego, CA: Academic Press) 195–257.

[B14] Hidalgo C. T. R. Tan E. S. H. Verlegh P. W. J. (2015). The social sharing of emotion (SSE) in online social networks: a case study in live journal. *Comput. Hum. Behav.* 52 364–372. 10.1016/j.chb.2015.05.009

[B15] Hoffmann F. Koehne S. Steinbeis N. Dziobek I. Singer T. (2016). Preserved self-other distinction during empathy in autism is linked to network integrity of right supramarginal gyrus. *J. Autism. Dev. Disord.* 46 637–648. 10.1007/s10803-015-2609-0 26476740

[B16] Hyatt C. J. Calhoun V. D. Pearlson G. D. Assaf M. (2015). Specific default mode subnetworks support mentalizing as revealed through opposing network recruitment by social and semantic FMRI tasks. *Hum. Brain Mapp.* 36 3047–3063. 10.1002/hbm.22827 25950551 PMC6869394

[B17] Ito T. A. Larsen J. T. Smith N. K. Cacioppo J. T. (1998). Negative information weighs more heavily on the brain: the negativity bias in evaluative categorizations. *J. Pers. Soc. Psychol.* 75 887–900. 9825526 10.1037//0022-3514.75.4.887

[B18] Java A. Song X. Finin T. Tseng B. (2007). Why we twitter: understanding microblogging usage and communities. *Paper presented at the Proceedings of the 9th WebKDD and 1st SNA-KDD 2007 Workshop on Web Mining and Social Network Analysis* San Jose, CA.

[B19] Johnson M. K. Raye C. L. Mitchell K. J. Touryan S. R. Greene E. J. Nolen-Hoeksema S. (2006). Dissociating medial frontal and posterior cingulate activity during self-reflection. *Soc. Cogn. Affect. Neurosci.* 1 56–64. 10.1093/scan/nsl004 18574518 PMC2435374

[B20] Kanai R. Bahrami B. Roylance R. Rees G. (2011). Online social network size is reflected in human brain structure. *Proc. Biol. Sci.* 279 1327–1334. 10.1098/rspb.2011.1959 22012980 PMC3282379

[B21] Kanske P. Bockler A. Trautwein F. M. Singer T. (2015). Dissecting the social brain: Introducing the EmpaToM to reveal distinct neural networks and brain-behavior relations for empathy and theory of mind. *Neuroimage* 122 6–19. 10.1016/j.neuroimage.2015.07.082 26254589

[B22] Kelley W. M. Macrae C. N. Wyland C. L. Caglar S. Inati S. Heatherton T. F. (2002). Finding the self? An event-related fMRI study. *J. Cogn. Neurosci.* 14 785–794. 10.1162/08989290260138672 12167262

[B23] Kennerley S. W. Walton M. E. (2011). Decision making and reward in frontal cortex: complementary evidence from neurophysiological and neuropsychological studies. *Behav. Neurosci.* 125 297–317. 10.1037/a0023575 21534649 PMC3129331

[B24] Kwak H. Lee C. Park H. Moon S. (2010). What is Twitter, a social network or a news media? *Paper Presented at the Proceedings of the 19th International Conference on World Wide Web* Raleigh, NC.

[B25] Lamm C. Decety J. Singer T. (2011). Meta-analytic evidence for common and distinct neural networks associated with directly experienced pain and empathy for pain. *Neuroimage*, 54 2492–2502. 10.1016/j.neuroimage.2010.10.014 20946964

[B26] Lamm C. Silani G. Singer T. (2015). Distinct neural networks underlying empathy for pleasant and unpleasant touch. *Cortex* 70 79–89. 10.1016/j.cortex.2015.01.021 25725510

[B27] Mars R. B. Sallet J. Schüffelgen U. Jbabdi S. Toni I. Rushworth M. F. S. (2012). Connectivity-based subdivisions of the human right “temporoparietal junction area”: evidence for different areas participating in different cortical networks. *Cereb. Cortex* 22 1894–1903. 10.1093/cercor/bhr268 21955921

[B28] Meshi D. Mamerow L. Kirilina E. Morawetz C. Margulies D. S. Heekeren H. R. (2016). Sharing self-related information is associated with intrinsic functional connectivity of cortical midline brain regions. *Sci. Rep.* 6:22491 10.1038/srep22491 PMC4780087 26948055

[B29] Neuberg S. L. Kenrick D. T. Schaller M. (2011). Human threat management systems: self-protection and disease avoidance. *Neurosci. Biobehav. Rev.* 35 1042–1051. 10.1016/j.neubiorev.2010.08.011 20833199 PMC3024471

[B30] O’Donnell M. B. Falk E. B. Lieberman M. D. (2015). Social in, social out: how the brain responds to social language with more social language. *Commun. Monogr.* 82 31–63. 10.1080/03637751.2014.990472 27642220 PMC5026191

[B31] Ohira H. Winton W. M. Oyama M. (1998). Effects of stimulus valence on recognition memory and endogenous eyeblinks: further evidence for positive-negative asymmetry. *Pers. Soc. Psychol. Bull.* 24 986–993. 10.1177/0146167298249006

[B32] Otto A. K. Laurenceau J.-P. Siegel S. D. Belcher A. J. (2015). Capitalizing on everyday positive events uniquely predicts daily intimacy and well-being in couples coping with breast cancer. *J. Fam. Psychol.* 29 69–79. 10.1037/fam0000042 25528074 PMC5407905

[B33] Phillips M. L. Young A. W. Senior C. Brammer M. Andrew C. Calder A. J. (1997). A specific neural substrate for perceiving facial expressions of disgust. *Nature* 389 495–498. 10.1038/39051 9333238

[B34] Poels K. Dewitte S. (2006). *How to Capture the Heart? Reviewing 20 Years of Emotion Measurement in Advertising*. Rochester, NY: Social Science Research Network.

[B35] Pratto F. John O. P. (1991). Automatic vigilance: the attention-grabbing power of negative social information. *J. Pers. Soc. Psychol.* 61 380–391. 1941510 10.1037//0022-3514.61.3.380

[B36] Schilbach L. Wilms M. Eickhoff S. B. Romanzetti S. Tepest R. Bente G. (2010). Minds made for sharing: initiating joint attention recruits reward-related neurocircuitry. *J. Cogn. Neurosci.* 22 2702–2715. 10.1162/jocn.2009.21401 19929761

[B37] Schurz M. Radua J. Aichhorn M. Richlan F. Perner J. (2014). Fractionating theory of mind: a meta-analysis of functional brain imaging studies. *Neurosci. Biobehav. Rev.* 42 9–34. 10.1016/j.neubiorev.2014.01.009 24486722

[B38] Strombach T. Weber B. Hangebrauk Z. Kenning P. Karipidis II Tobler P. N. Kalenscher T. (2015). Social discounting involves modulation of neural value signals by temporoparietal junction. *Proc. Natl. Acad. Sci. U.S.A.* 112 1619–1624. 10.1073/pnas.1414715112 25605887 PMC4321268

[B39] Subramani M. R. Rajagopalan B. (2003). Knowledge-sharing and influence in online social networks via viral marketing. *Commun. ACM* 46 300–307. 10.1145/953460.953514

[B40] Tacikowski P. Brechmann A. Nowicka A. (2013). Cross-modal pattern of brain activations associated with the processing of self- and significant other’s name. *Hum. Brain Mapp.* 34(9) 2069–2077. 10.1002/hbm.22048 22431327 PMC6869889

[B41] Takahashi H. Kato M. Matsuura M. Mobbs D. Suhara T. Okubo Y. (2009). When your gain is my pain and your pain is my gain: neural correlates of envy and schadenfreude. *Science* 323 937–939. 19213918 10.1126/science.1165604

[B42] Tang H. H. Mai X. Q. Wang S. Zhu C. Z. Krueger F. Liu C. (2016). Interpersonal brain synchronization in the right temporo-parietal junction during face-to-face economic exchange. *Soc. Cogn. Affect. Neurosci.* 11 23–32. 10.1093/scan/nsv092 26211014 PMC4692317

[B43] Wagner U. Galli L. Schott B. H. Wold A. van der Schalk J. Manstead A. S. R. (2015). Beautiful friendship: social sharing of emotions improves subjective feelings and activates the neural reward circuitry. *Soc. Cogn. Affect. Neurosci.* 10 801–808. 10.1093/scan/nsu121 25298009 PMC4448023

[B44] Welborn B. L. Lieberman M. D. Goldenberg D. Fuligni A. J. Galvan A. Telzer E. H. (2016). Neural mechanisms of social influence in adolescence. *Soc. Cogn. Affect. Neurosci.* 11 100–109. 10.1093/scan/nsv095 26203050 PMC4692320

[B45] Yan C.-G. Cheung B. Kelly C. Colcombe S. Craddock R. C. Di Martino A. Milham M. P. (2013). A comprehensive assessment of regional variation in the impact of head micromovements on functional connectomics. *Neuroimage* 76 183–201. 10.1016/j.neuroimage.2013.03.004 23499792 PMC3896129

[B46] Yan C. -G. Wang X. -D. Zuo X. -N. Zang Y. -F. (2016). DPABI: data processing & analysis for (Resting-State) brain imaging. *Neuroinformatics* 14(3) 339–351. 10.1007/s12021-016-9299-4 27075850

[B47] Yan C.-G. Zang Y.-F. (2010). DPARSF: A MATLAB Toolbox for “Pipeline” data analysis of resting-state fMRI. *Front. Syst. Neurosci.* 4:13 10.3389/fnsys.2010.00013 PMC2889691 20577591

[B48] Zerubavel N. Bearman P. S. Weber J. Ochsner K. N. (2015). Neural mechanisms tracking popularity in real-world social networks. *Proc. Natl. Acad. Sci. U.S.A.* 112(49) 15072–15077. 10.1073/pnas.1511477112 26598684 PMC4679039

